# Balloon inflation for a mechanical tricuspid valve thrombosis: case report

**DOI:** 10.1093/ehjcr/ytaf367

**Published:** 2025-07-31

**Authors:** Shaw Hua (Anthony) Kueh, Mark Webster

**Affiliations:** Green Lane Cardiovascular Services/Cardiology Department, Auckland City Hospital, Te Toka Tumai Auckland, Health New Zealand—Te Whatu Ora, Park Road, Grafton, Auckland 1142, New Zealand; Green Lane Cardiovascular Services/Cardiology Department, Auckland City Hospital, Te Toka Tumai Auckland, Health New Zealand—Te Whatu Ora, Park Road, Grafton, Auckland 1142, New Zealand

**Keywords:** Mechanical valve, Valve intervention, Cardiac computed tomography, Thrombolysis, Case report

## Abstract

**Background:**

Mechanical valve thrombosis (MVT) is rare but life-threatening complication. While the clinical guideline suggests that thrombolysis for high-risk surgical candidates should be considered, the European guideline does not differentiate between left- and right-sided MVTs while the American guideline only made specific recommendations for left-sided MVT. Furthermore, the American guideline suggests that percutaneous intervention for left-sided MVT may be considered. Both clinical guidelines did not refer to percutaneous intervention for right-sided MVT.

**Case summary:**

A 52-year-old woman with three mechanical valve replacements for rheumatic heart disease presented with subacute onset of symptoms of right-sided heart failure. Transthoracic echocardiogram (TTE) demonstrated a high transvalvular gradient across the tricuspid mechanical valve which is new from TTE 3 years prior. Further imaging confirmed thrombosis of the anterior leaflet of the tricuspid mechanical valve. Four cycles of ultra-low-dose thrombolysis and one standard-dose thrombolysis failed to restore normal valve function. Surgery was considered prohibitively high-risk, and the patient was left with either palliation or percutaneous intervention. Percutaneous mechanical valve balloon inflation was undertaken with subsequent improvement in transvalvular gradient and leaflet motion.

**Discussion:**

There is limited data on percutaneous intervention for right-sided MVT with only one other case reported to date. In patients with prohibitively high surgical risk, thrombolysis should be considered as per the clinical guidelines. However, in ∼1 in 10 patients, thrombolysis fails to restore normal valve function. Percutaneous intervention may be an alternative option.

Learning points

- Mechanical valve thrombosis (MVT) is a rare but potentially life-threatening complication. Current clinical guidelines suggest thrombolysis should be considered in those who are high-risk surgical candidates.
- In ∼1 in 10 patients, thrombolysis fails to restore valve function.
- Although the clinical guidelines made no reference to percutaneous intervention for right-sided MVT, our case suggests that this can be done safely and may be a reasonable alternative to palliation.

## Introduction

The 2021 European Society of Cardiology (ESC) guideline favours surgery for those with obstructive valve thrombosis in critically unwell patients without significant comorbidities (Class I, LOC B) and those with large (>10 mm) non-obstructive prosthetic thrombus complicated by embolism (Class IIa, LOC B).^1^ Thrombolysis is reserved for those with very high surgical risk, in centres where surgery is not available and for those with a right-sided prosthesis (Class IIa, LOC C). In contrast, the 2020 American College of Cardiology/American Heart Association Joint Committee (ACC/AHA) guideline gave a Class I indication for thrombolysis or emergency surgery for those with symptomatic left-sided mechanical valve thrombosis (MVT) but does not address right-sided MVT.^2^

Thrombolysis fails to restore valve function in ∼10% of patients.^3^ These patients are limited to either high-risk operation or palliation. Balloon inflation of mechanical valve has been described for left-sided MVT in case series,^4–6^ however, there has only been one case report for tricuspid MVT in a patient with Ebstein anomaly.^7^ We described our considered approach in a patient with rheumatic heart valve disease with previous triple mechanical valve replacement who presented with tricuspid MVT. After failing thrombolysis, she underwent percutaneous balloon inflation of the right-sided mechanical valve with subsequent improvement in transvalvular gradient and valve function.

## Summary figure

**Table:** 

Day 0	Transthoracic echocardiogram **Tricuspid mechanical valve**—**disc motion not well seen. Transvalvular mean gradient 8 mmHg**. **Normal LV size with low-normal LV function. Normal RV size with mild systolic impairment. AV: mean gradient 31 mmHg, DDI 0.42. MV: mean gradient 5 mmHg**.
	Non-contrast CT TV anterior leaflet closed and immobile. AV and MV leaflet normal function.
Day 1	Alteplase 1 mg/h for 25 h
Day 4	Transthoracic echocardiogram Tricuspid transvalvular mean gradient 7 mmHg at heart rate of 87 b.p.m. Disc motion not well seen.
	Alteplase 1 mg/h for 25 h
Day 5	Transthoracic echocardiogram Tricuspid transvalvular mean gradient 7 mmHg at heart rate of 85 b.p.m. Disc motion not well seen.
Day 6	Alteplase 1 mg/h for 25 h
Day 7	Transthoracic echocardiogram Tricuspid transvalvular mean gradient 4 mmHg at heart rate 78–87 b.p.m. Disc motion not well seen.
Day 8	Alteplase 1 mg/h for 25 h
Day 10	Transthoracic echocardiogram Tricuspid transvalvular mean gradient 5 mmHg at heart rate of 76 b.p.m.
	Cardiac CT Severe restriction of the anterior leaflet of the mechanical tricuspid valve. No large valvular or intra-cardiac thrombus seen.
Day 11	Alteplase 10 mg over 2 min then 90 mg over 90 min
Day 12	Transthoracic echocardiogram Tricuspid transvalvular mean gradient 7 mmHg with heart rate of 75 b.p.m.
12/06	Cardiac CT Anterior leaflet of the mechanical tricuspid valve remains immobile.
Day 13	Mechanical balloon valvuloplasty Stuck anterior leaflet tricuspid mechanical valve easily crossed with AL1 catheter and various wires. Any wire across resulted in leaflet motion. Ballooned with 6 mm balloon, 8 mm balloon, and two 6 mm balloons. Heparin was initially sub-therapeutic. Required 10 000 U for ACT to reach therapeutic range. Leaflet re-stuck once wire removed. Good haemostasis.
13/06	Transthoracic echocardiogram Tricuspid transvalvular mean gradient 6 mmHg.
Day 17	Diagnostic fluoroscopy Anterior leaflet of mechanical tricuspid valve opens 1:3—improved from last week but still not fully mobile.
Day 19	Cardiac CT The anterior leaflet of the tricuspid mechanical valve is now mobile with some restriction. The leaflet does not appear to close fully. The posterior leaflet is now severely restricted with minimal leaflet opening seen.
Day 20	Transthoracic echocardiogram Anterior leaflet appears to function normally. The posterior leaflet appears to close intermittently (∼1:5). Tricuspid transvalvular mean gradient of 7 mmHg.
Day 24	Transthoracic echocardiogram Delayed opening of the posterior leaflet on occasions and remains close in 1 in 4 heartbeat. Tricuspid transvalvular mean gradient of 7 mmHg at heart rate of 80 b.p.m.

## Case summary

A 52-year-old Nepalese woman received 18 mm ATS mechanical aortic valve, 28 mm St Jude mechanical mitral valve, and 27 mm St Jude mechanical tricuspid valve replacements in 2014 for rheumatic heart valve disease. In 2016, she presented with subacute heart failure and was found to have constrictive pericarditis requiring pericardiectomy. A year later, she re-presented with right-sided heart failure due to subacute tricuspid valve thrombosis, for which she had urgent redo tricuspid valve replacement. Severe sternal adhesions were encountered, and an organized thrombus was found on the 27 mm St Jude mechanical tricuspid valve. This was replaced with a 27 mm ATS mechanical valve. She recovered well and remained compliant with her anticoagulation.

On this occasion, seven years later, she re-presented with a 3-month history of intermittent nausea, abdominal distension, and exertional dyspnoea. An echocardiogram showed an increased tricuspid transvalvular gradient of 8 mmHg, compared with 4 mmHg 3 years earlier. No obvious thrombus was seen. On admission, she was haemodynamically stable (heart rate 88 b.p.m., BP 151/89 mmHg, Sat 98% on air). Her jugular venous pressure was elevated at 5 cm with associated mild bilateral pedal oedema and a pulsatile liver. She had bibasal crepitation on chest auscultation. Heart sounds were dual with mechanical S1 and S2. There is a 2/6 ejection systolic murmur audible in the aortic position. No diastolic murmur or split S2 was audible. Her international normalized ratio (INR) was 2.1. Her renal function was stable (creatinine 128 umol/L). Her medical history included chronic atrial fibrillation, type 2 diabetes with nephropathy, previous gastritis, and proctitis with bleeding in 2021.

Given the subacute nature of the presentation, the interval increase in the tricuspid transvalvular gradient and sub-therapeutic INR level, MVT was thought to be most likely. Pannus formation was possible but thought to be less likely.

**Figure 1:**
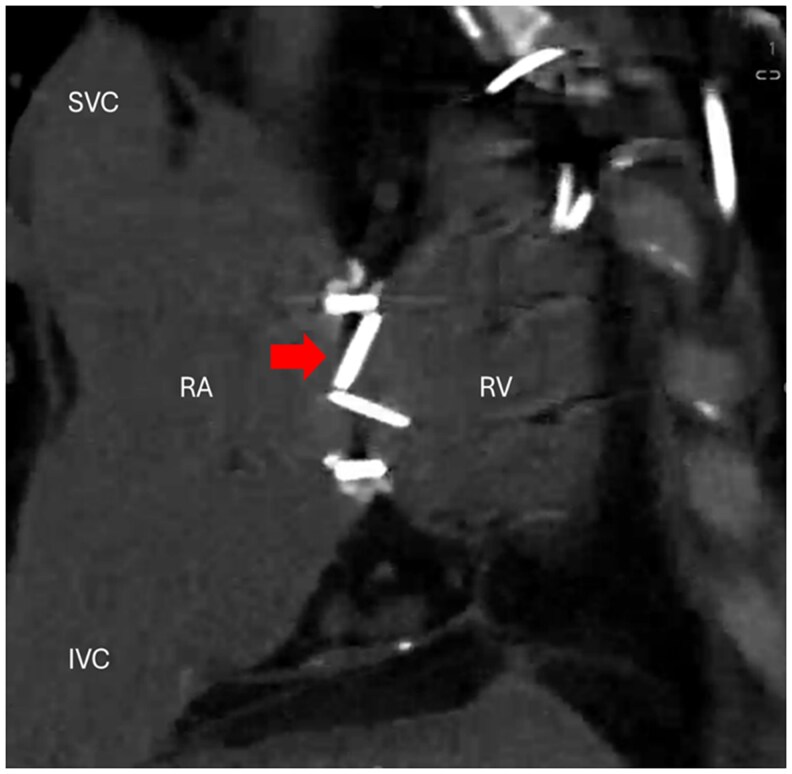
Non-contrast gated cardiac computed tomography showing the immobile anterior leaflet of the 27 mm St Jude bileaflet mechanical valve (arrow). SVC, superior vena cava; IVC, inferior vena cava; RA, right atrium; RV, right ventricle.

A non-contrast gated cardiac CT found that the anterior leaflet of the mechanical tricuspid valve was immobile (*Figure 1*). The aortic and mitral mechanical valves were mobile without restriction. The echocardiogram showed normal left ventricle with low-normal systolic function. The right ventricle was normal in size with mild systolic impairment.

**Figure 2:**
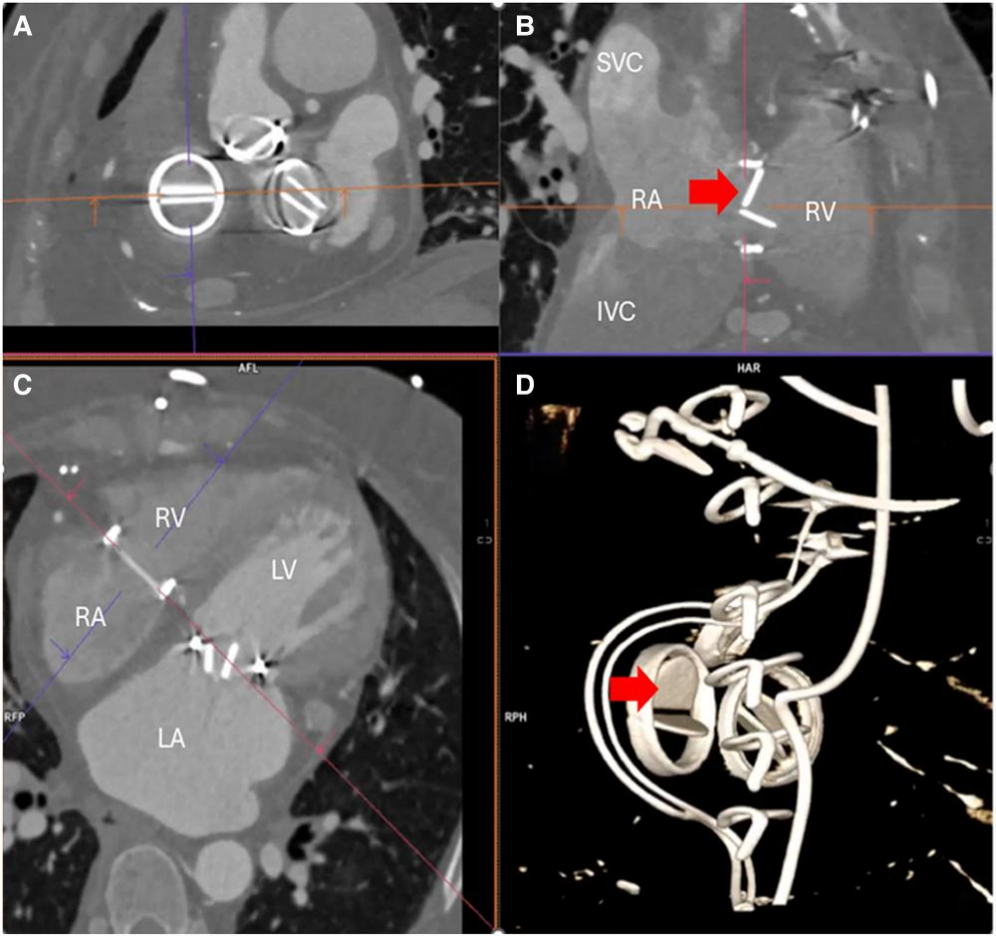
Contrast-enhanced gated cardiac computed tomography after four cycles of ultra-low-dose thrombolysis. The anterior leaflet of the mechanical valve remained immobile in diastole (arrow), and no atrial or valvular thrombus was seen. *Panel A* shows the enface view, and *Panel B* shows the cross-section of the 27 mm St Jude mechanical tricuspid valve. *Panel C* shows the four-chamber view. *Panel D* is the volume rendering showing the immobile anterior leaflet of the tricuspid mechanical valves (arrow), pacing wires, and sternal wires. LV, left ventricle; LA, left atrium.

Redo surgery was thought to be prohibitive risk and she went on to receive four cycles of ultra-low-dose thrombolysis^2,3^ (alteplase at 1 mg/h for 25 h) with 6 h of UFH infusion between each cycle. Although serial echocardiograms suggested an improvement in the tricuspid transvalvular gradient, a repeat CT demonstrated that the anterior leaflet remained immobile (*Figure 2*, Supplementary material online, *Video CT-1*). A further standard dose of thrombolysis was then given as per the ESC guideline^1^ with no effect.

**Figure 3:**
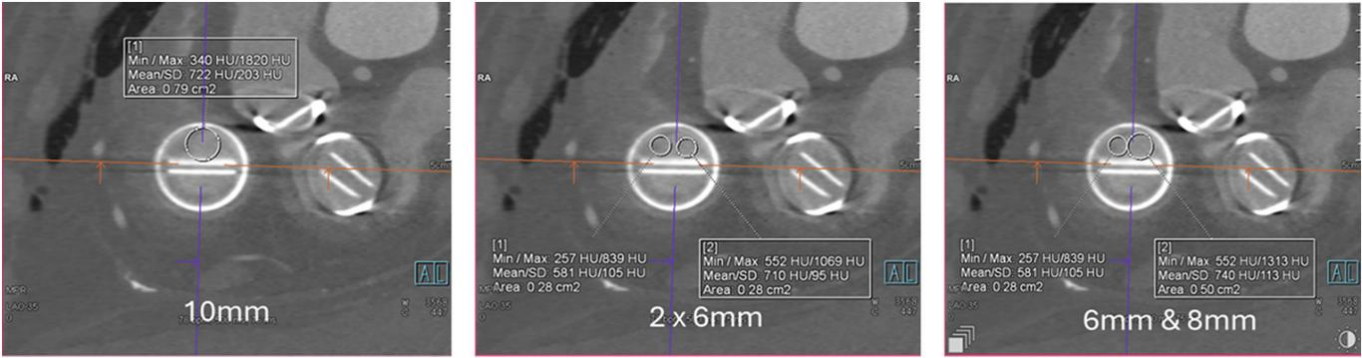
Enface view of the mechanical tricuspid valve. The circle drawn simulates the percutaneous balloon inflated to nominal size in the anterior orifice of the mechanical valve with a single 10 mm balloon (left panel), two 6 mm balloon side by side (middle panel), and a 6 mm balloon and an 8 mm balloon side by side (right panel).

**Figure 4:**
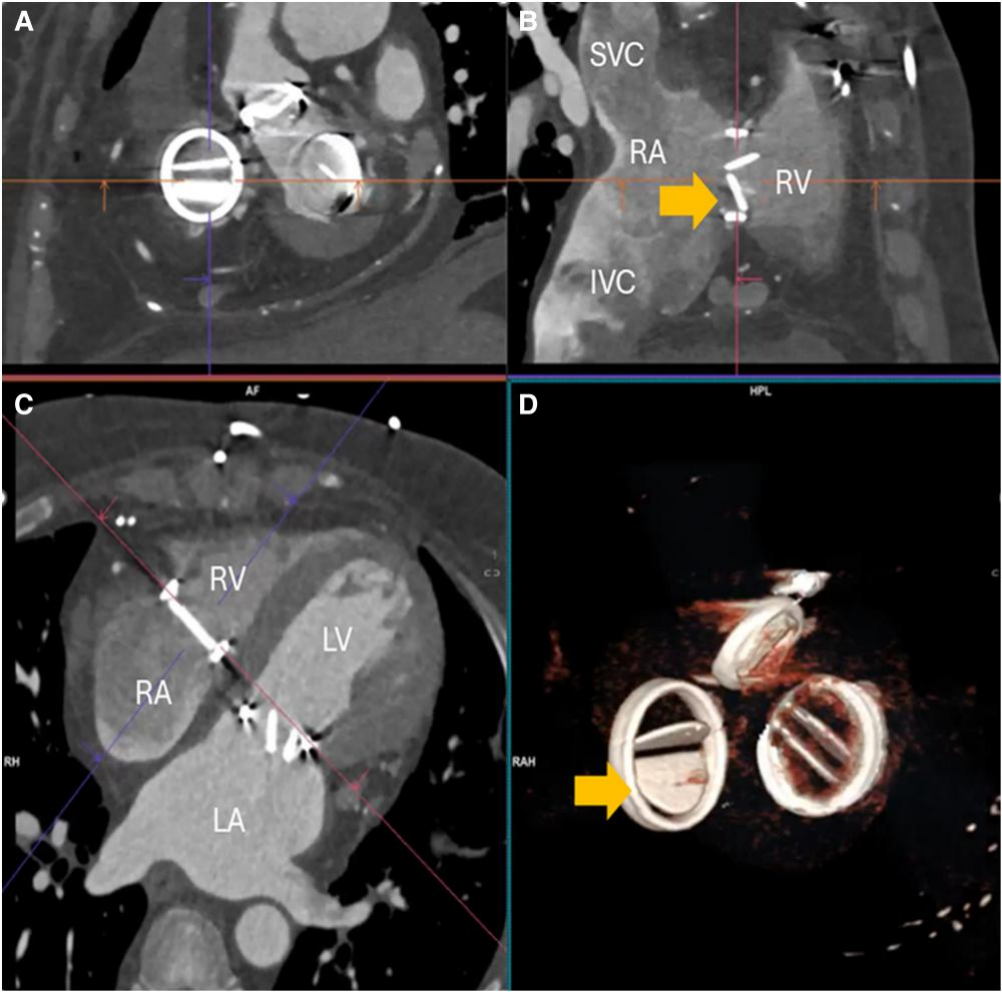
Contrast-enhanced gated cardiac computed tomography at diastole post-thrombolysis and percutaneous intervention and on therapeutic anticoagulation. The anterior leaflet was open, but the posterior leaflet was now immobile at diastole (arrow on *Panels B* and *D*).

Having failed thrombolysis, percutaneous intervention was undertaken. The immobile anterior leaflet was easily crossed with a catheter and wire. Once crossed, the leaflet motion returned but became immobile again once the wire was removed. Balloon inflation in the anterior orifice of the tricuspid mechanical valve was undertaken, initially with a 6 mm balloon, then an 8 mm balloon (see Supplementary material online, *Video Cath-1*), and finally two 6 mm balloons (*Figure 3*, Supplementary material online, *Video Cath-2*). Balloon selection was guided by CT imaging (*Figure 4*). At the end of the procedure, the anterior leaflet opened intermittently. The patient was recommenced back on therapeutic UFH. Post-procedural transthoracic echocardiogram showed a marginal improvement in the transvalvular gradient. Follow-up diagnostic fluoroscopy 5 days later found the anterior leaflet continued to open intermittently (see Supplementary material online, *Video Cath-3*). Follow-up cardiac CT 7 days later showed that the anterior leaflet motion had improved with some restriction, however the posterior leaflet had become immobile (see Supplementary material online, *Video CT-2*). The patient remained clinically stable with NYHA Class I–II symptoms. The patient was re-established back on warfarin therapy with a target INR of 3.5.

**Figure 5:**
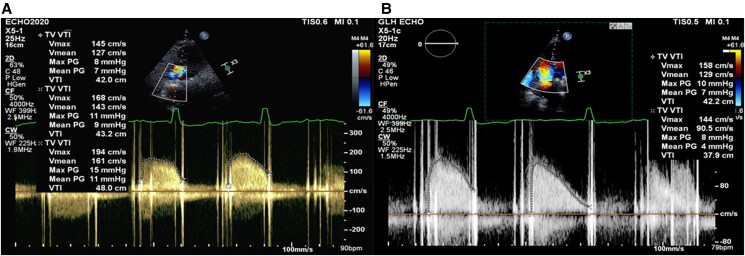
Transvalvular gradient of 27 mm St Jude mechanical tricuspid valve at presentation (*A*) and at 2-month follow-up (*B*). The average transvalvular mean gradient was 8 mmHg in atrial fibrillation with a maximum mean gradient of up to 11 mmHg at presentation. The continuous wave (CW) Doppler exhibits a parabolic profile and long pressure half-time consistent with severe valvular stenosis (*A*). At follow-up, there was a reduction in the average transvalvular mean gradient of 6 mmHg, return of the normal triangular CW profile, and a reduction in pressure half-time (*B*).

A follow-up echocardiogram 2 months later showed an improvement in the tricuspid transvalvular gradient (*Figure 5*). The anterior leaflet was mobile while the posterior leaflet opened intermittently. At 6 month follow-up, the patient had not suffered any major bleeding or clinical evidence of thromboembolism.

## Discussion

Current guidelines do not provide recommendations for the role of percutaneous intervention in MVT but suggest that this can be considered.^1,2^ To date, there have only been case reports and one small case series of left-sided mechanical balloon inflation.^4^ For right-sided MVT, there has only been one case of percutaneous intervention on the tricuspid mechanical valve reported previously.^7^ A 62-year-old female with 31 mm Carbomedic bileaflet MHV replacement for Ebstein’s anomaly presented in cardiogenic shock. Marked improvement in the transvalvular gradient was described (mean gradient of 15–4 mmHg). To our knowledge, our case represents the first reported case of percutaneous intervention of a tricuspid mechanical valve in a patient with multiple mechanical valves for rheumatic heart valve disease. The percutaneous balloon inflation of the anterior mechanical leaflet resulted in an improvement in the transvalvular gradient and valvular function.

### Thrombolysis dosing

The ESC guideline recommends standard thrombolytic regimens,^1^ whereas the ACC/AHA guideline suggests using either a slow- or low-dose tPA infusion.^2^ Low-dose thrombolysis (25 mg over 6 h) using tPA was shown to achieve restoration of valve function in 85% of patients with a significantly lower rate of complications compared to the standard or reduced dosing (10% vs. 37% with rapid streptokinase, 24% with slow streptokinase, 33% with 100 mg tPA, and 30% with 50 mg tPA infusion over 6 h).^8^ Further dose reduction with tPA of 25 mg over 25 h (ultra-low-dose) had a success rate of 90% and fewer complications (mortality 1%; major non-fatal complication 3%).^9^ In a subsequent multi-centre registry using the ultra-low-dose thrombolysis and at a median dose of 59 mg of tPA, Özkan *et al.*^3^ demonstrated an overall success of thrombolysis of 90% with low rate of complications (embolic events 2% vs. 5%; bleeding 8% vs. 16%; 3-month mortality 2% vs. 19%). In our patient, initial ultra-low-dose thrombolysis followed by standard-dose thrombolysis failed to restore normal valve function.

## Conclusion

A right-sided mechanical valve is at higher risk of thrombosis compared to the left. There is, presently, insufficient data to guide the appropriate management of right-sided MVT, and decisions favouring surgery or thrombolysis need to be individualized. For those who failed thrombolysis and surgery is prohibitively high-risk, percutaneous balloon inflation may facilitate restoration of valve function.

## Lead author biography


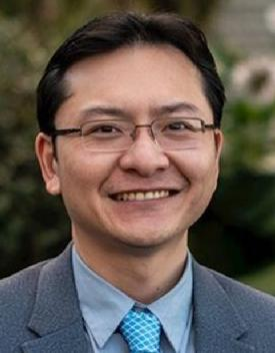


Dr Shaw Hua (Anthony) Kueh is a Cardiologist at Auckland City Hospital, New Zealand. He graduated from the Dunedin School of Medicine at the University of Otago and completed his Cardiology training at Green Lane Cardiovascular Services. He was supported by the New Zealand Heart Foundation to undergo fellowship training in cardiac CT and MRI at St Paul’s Hospital, Vancouver, Canada. He has a particular interest in structural imaging and is a mentor and supervisor for trainees of the Royal College of Physicians.

## Supplementary Material

ytaf367_Supplementary_Data

## Data Availability

The data underlying this article cannot be shared publicly due to privacy and ethical reasons. The data will be shared on reasonable request to the corresponding author.

## Notes

**Consent:** Written consent was obtained from the patient for the publication of this study and accompanying images. Patient consent was compliant with the COPE guidelines.
